# A BRAF mutation-associated gene risk model for predicting the prognosis of melanoma

**DOI:** 10.1016/j.heliyon.2023.e15939

**Published:** 2023-05-02

**Authors:** Xiang Huang, Wanrong Gou, Qinxian Song, Yan Huang, Chunlei Wen, Xue Bo, Xian Jiang, Jianguo Feng, Hong Gao

**Affiliations:** a Department of Anesthesiology, The Affiliated Hospital of Guizhou Medical University, Guiyang, 550004, Guizhou, China; b Anesthesiology and Critical Care Medicine Key Laboratory of Luzhou, Department of Anesthesiology, The Affiliated Hospital of Southwest Medical University, Luzhou, 646000, Sichuan, China; c Department of Dermatology, Suining First People's Hospital, Suining, 629000, Sichuan, China

**Keywords:** Melanoma, BRAF mutation, Prediction model, Immune checkpoint, Prognosis

## Abstract

BRAF mutation plays an important role in the pathogenesis and progression of melanoma and is correlated to the prognosis of melanoma patients. However, fewer studies have attempted to develop a BRAF mutation-associated gene risk model for predicting the prognosis of melanoma. The current research explores BRAF mutation-related biological features in melanoma and establishes a prognostic signature. First, we identified three significantly enriched KEGG pathways (glycosphingolipid biosynthesis - ganglio series, ether lipid metabolism, and glycosaminoglycan biosynthesis - keratan sulfate) and corresponding genes in the BRAF mutant group by gene set enrichment analysis. We then developed a prognostic signature based on 7 BRAF-associated genes (PLA2G2D, FUT8, PLA2G4E, PLA2G5, PLA2G1B, B3GNT2, and ST3GAL5) and assessed its prediction accuracy using ROC curve analysis. Finally, the nomogram was established according to the prognostic signature and independent clinical characteristics to predict the survival of melanoma patients. Furthermore, we found higher proportions of naive B cells, plasma cells, CD8 T cells, CD4 memory-activated T cells, and regulatory T cells in the low-risk group. Whereas lower proportions of M0, M1, and M2 macrophages and resting NK cells were observed in the high-risk group. The analysis also showed a significantly higher expression of immune checkpoint molecules (PD-1, PD-L1, CTLA4, BTLA, CD28, CD80, CD86, HAVCR2, ICOS, LAG3, and TIGIT) in the low-risk group. Our results provide novel insights into the effect of BRAF mutation on melanoma growth and indicate a promising direction toward immunotherapy and precision medicine in melanoma patients.

## 1 Introduction

Melanoma is a malignant tumor originating from melanocytes with an increasing incidence worldwide and accounts for most skin cancer–related deaths [1]. Although surgical resection has been proven effective for early-stage melanoma patients with favorable results, patients in the late-stage and with regional metastases have a poor prognosis [2]. Currently, there are many established treatments for melanoma, including surgery, systemic treatment, radiotherapy, chemotherapy, adjuvant therapy, combined targeted therapy, and immunotherapy [3]. And a recent study reported that functionalized exosome platform could provide a promising strategy for targeted therapy of malignant melanoma [4]. However, despite recent advances in treatment strategies, melanoma remains a severe public health threat.

Mutation in BRAF, a serine-threonine kinase involved in the mitogen-activated protein kinase signal transduction pathway, is one of the most common oncogenic mutations in melanoma [5,6]. The substitution of valine with glutamate at codon 600 (V600E) accounts for approximately 90% of the BRAF mutations in melanoma, resulting in constitutive kinase activity and unregulated cell proliferation [7,8]. Previous studies have shown that dabrafenib or vemurafenib, a selective BRAF inhibitor, significantly improves progression-free survival (PFS), clinical response rate (RR), and overall survival (OS) compared to chemotherapy in BRAF-mutant melanoma patients [9,10]. Furthermore, combining a BRAF inhibitor with a MEK inhibitor significantly improved OS in BRAF-mutant metastatic melanoma patients without an increase in overall toxicity [11]. Although the BRAF inhibitor leads to initial tumor regression, most melanoma patients experience recurrence within one year [9,10,12]. The clinical success of BRAF inhibitors in long-term treatment is limited due to drug resistance. Thus, it is critical to investigate further the effects of BRAF on the pathogenesis of melanoma. Several prognostic prediction models for melanoma have been constructed based on hypoxia score [13], ferroptosis-related gene [14], immune-related gene [15], glycolysis-related gene [16], and pyroptosis-related lncRNA [17]. However, the prediction ability of these prognostic models needs further evaluation due to poor predictive performance in the validation cohort (AUC = 0.628) [13], the absence of time-dependent receiver operative characteristic (ROC) analysis [14,15], or the lack of a validation cohort [16,17]. To the best of our knowledge, there was no prediction model based on BRAF mutation status in melanoma.

Immune checkpoint inhibitors (ICIs) targeting PD-1 (nivolumab and pembrolizumab) and CTLA-4 (ipilimumab), approved for use in advanced unresectable or metastatic melanoma patients, possess impressive antitumor effects. For example, a study reported that ipilimumab improved OS in patients with previously treated metastatic melanoma compared with glycoprotein 100 (gp100) alone [18]. Another phase III trial noted that a combination of ipilimumab and dacarbazine significantly increased the 5-year survival rate for melanoma patients compared with a placebo plus dacarbazine [19]. Notably, after treatment with nivolumab and ipilimumab, melanoma patients with BRAF mutant had significantly higher OS at five years than those without BRAF mutation (60% vs. 48%), suggesting that the immune microenvironment of BRAF-mutant melanoma may be distinct from that of wild type melanoma. Further, BRAF mutation can activate the mitogen-activated protein kinase (MAPK) signaling pathway. In addition, another study showed that activation of the MAPK signaling pathway is critical for cancer-immune evasion in BRAF-mutant melanoma cells [20]. Therefore, the clinical efficacy of ICIs may vary due to the distinct immune microenvironment in melanoma patients with different BRAF mutation status. And thus, melanoma patients will benefit from a better understanding of immune phenotypes connected to BRAF mutation, resulting in better therapy and improved outcomes. Subsequently, it is essential to clinically screen melanoma patients sensitive to ICIs for individualized treatment.

**Fig. 1:**
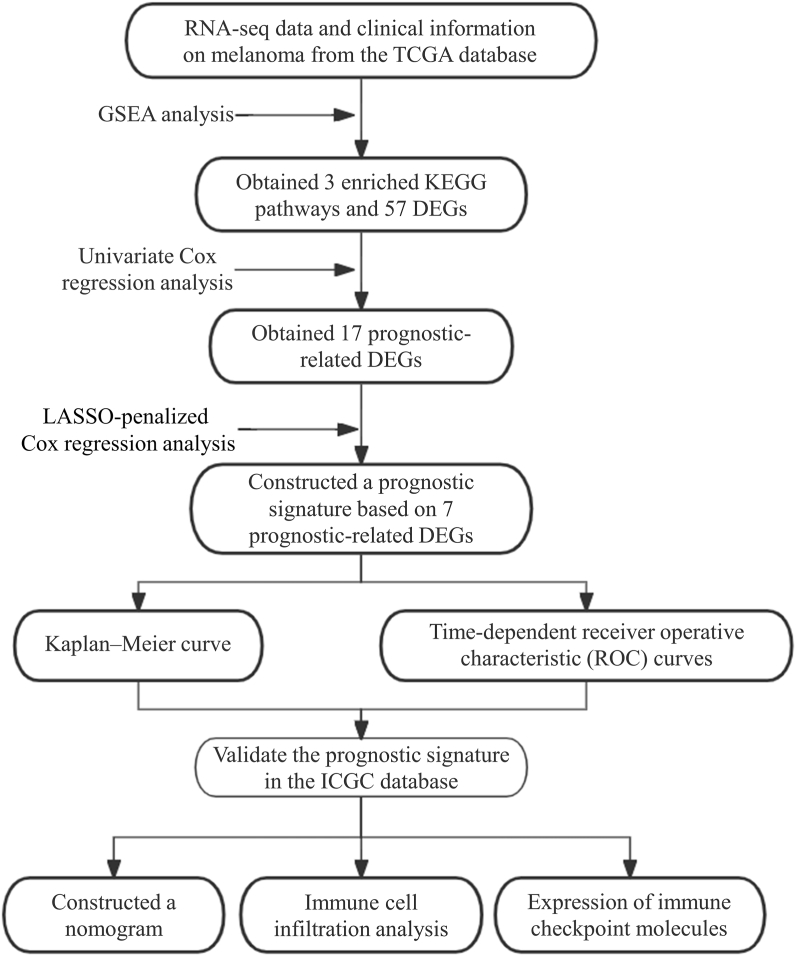
The schematic of the workflow for this study.

In this study, we obtained 3 enriched KEGG pathways and 57 differentially expressed genes (DEGs) by Gene set enrichment analysis (GSEA). Subsequently, Univariate cox regression analysis and LASSO-penalized Cox regression analysis were applied to construct a prognostic signature. ROC curve analysis was used to assess its prediction accuracy. We also validated the prediction accuracy of this prognostic signature in the International Cancer Genome Consortium (ICGC) cohort. Then, a nomogram was constructed based on risk scores and clinical factors. The workflow of the study is shown in Fig. 1. We explored BRAF-related biological features in melanoma and established a prognostic signature taking into consideration the clinical risk factors to precisely predict the probability of OS for melanoma patients. Furthermore, we also found the prognostic signature was related to immune cell infiltration and immune checkpoint. Our results may inform the clinical trial design to understand the potential mechanisms of drug resistance to BRAF inhibitors in melanoma patients. In addition, the selected genes may serve as biomarkers and potential therapeutic targets for melanoma.

## 2 Materials and methods

### 2.1 Data source

The RNA sequencing data and the corresponding clinical phenotypes of melanoma patients were obtained on 15 September 2022 from the Cancer Genome Atlas (TCGA) database (https://www.cancer.gov/) using the University of California at Santa Cruz (UCSC) Xena portal (http://xena.ucsc.edu/) [21]. The RNA sequencing data and relevant clinical datasheets for validation were downloaded from the ICGC database (ICGC, https://dcc.icgc.org/).

### 2.2 GSEA

The differences in KEGG pathways and corresponding genes between mutant and wild melanoma samples in the TCGA database were analyzed via GSEA (Version:4.2.3) [22]. The gene set databases c2.cp.kegg.v2022.1.Hs.symbols.gmt and c5.go.bp.v2022.1.Hs.symbols.gmt were used, and a threshold of *p-value* < 0.05 and FDR ≤0.25 were used to select significantly enriched pathways.

### 2.3 Prognostic signature establishment

Three significantly enriched KEGG pathways in the BRAF mutant group and 57 corresponding genes were obtained using GSEA. We then performed a univariate Cox regression analysis to assess the prognostic value of these genes. And the result showed that 20 of 57 BRAF-related genes were significantly associated with melanoma patients’ OS. Next, we performed lasso-penalized Cox regression analysis to select genes with the most significant prognostic value among the 20 genes. The optimal L1 penalty parameter was obtained by applying 10-fold cross-validation partial likelihood scores, and seven candidate genes were acquired. Next, we constructed the prognostic signature through the glmnet R package. The risk score of each melanoma patient was calculated by the following formula:

1 Riskscore=exprPLA2G2D×λPLA2G2D+exprFUT8×λFUT8+exprPLA2G4E×λPLA2G4E+exprPLA2G5×λPLA2G5+exprPLA2G1B×λPLA2G1B+exprB3GNT2×λB3GNT2+exprST3GAL5×λST3GAL5

The melanoma patients in TCGA or ICGC database were divided into low-risk and high-risk groups according to the optimal cutoff point, which was determined through the maxstat R package. The ability of prognostic signature to predict the OS of melanoma patients was evaluated based on Kaplan–Meier curve using the survival R package. ROC curves were applied to examine the prognostic ability of the prognostic signature by survival ROC R package.

### 2.4 Construction and evaluation of the nomogram

First, we performed a univariate Cox regression analysis to assess the association between clinical variables and OS. Then the multivariate Cox regression was conducted to identify independent prognostic factors of OS. The nomogram was established based on three independent prognostic factors to evaluate the probability of 3- and 5-year OS for melanoma patients. The calibration curves and concordance index (C-index) values confirm the predictive performance of the nomogram.

### 2.5 Immune cell infiltration analysis

The CIBERSORT and the LM22 immune cell signature were used to assess the changes in immune infiltrates between low-risk and high-risk groups. To estimate the response to immune checkpoint blockade therapy, we also examined the expression of the immune checkpoint molecules in the melanoma samples.

### 2.6 Statistics analysis

All statistical analyses were conducted using the statistical R software, version 4.2.1. Survival analysis was performed using the Kaplan-Meier survival curve and logrank test. Student's t-test was used to compare the difference between two groups. P < 0.05 was considered statistically significant.

## 3 Results

### 3.1 Identification of DEGs between melanoma samples with and without BRAF mutations

**Fig. 2:**
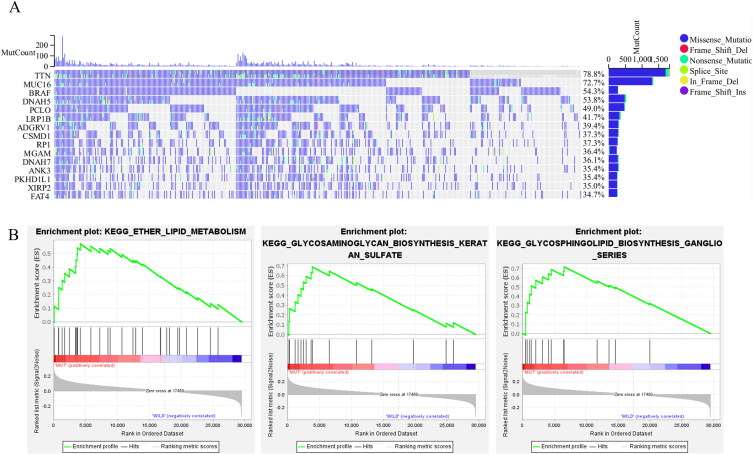
Identification of DEGs between melanoma samples with and without BRAF mutations. (A) Genomic landscape of melanoma and the mutational signatures in the TCGA cohort. (B) Significant enriched signaling pathways in BRAF^MUT^ melanomas patients compared with that in BRAF^WT^ melanoma patients.

BRAF is one of the major mutated genes in melanoma, according to the Cancer Genome Atlas (TCGA). Of the 467 patients in the TCGA SKCM cohort, 233 (54.3%) patients carried somatic mutations in BRAF (Fig. 2A). Following the discovery of BRAF^V600 E/K^ as an oncogenic driver of somatic mutation in melanomas, targeted therapy involving BRAF and MEK inhibitors was developed [9,10]. Given the vital implications of BRAF mutations in melanoma, GSEA was performed to analyze DEGs between the BRAF mutant and wild-type groups. The GSEA results showed that BRAF^MUT^ melanomas enriched three KEGG pathways: Glycosphingolipid biosynthesis – Ganglio series (NES = 1.983, size = 15), Ether lipid metabolism (NES = 1.874, size = 29), and Glycosaminoglycan biosynthesis – Keratan sulfate (NES = 1.845, size = 15) (*p-value* < 0.05, FDR <0.25; Fig. 2B). In contrast, no enrichment was found for any KEGG pathway in the wild-type group. Further, 57 genes corresponding to the three KEGG pathways were identified (Supplementary Table 1).

### 3.2 Prognostic signature based on DEGs

**Fig. 3:**
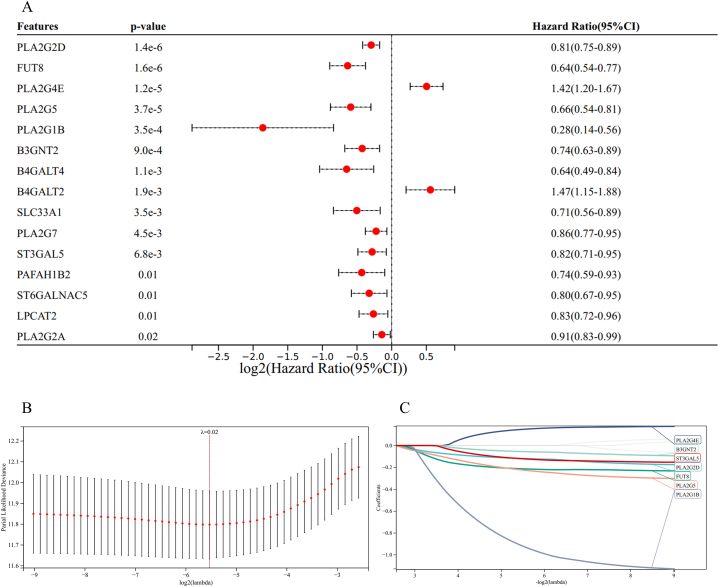
Construction of prognostic signature based on DEGs in the TCGA cohort. (A) Identification of prognostic DEGs by univariate Cox regression analysis. (B, C) Screening the most representative 7 genes in prognostic DEGs by LASSO-COX analysis.

Considering the importance of BRAF mutation in the treatment of melanoma, we performed a univariate Cox regression analysis to further evaluate the relationship between these 57 genes and the OS of melanoma patients. We identified a total of 19 genes that were significantly related to OS (Fig. 3A). To screen these genes with the most significant prognostic value, we applied LASSO-penalized Cox regression analysis, and a prognostic signature was constructed based on seven genes (Fig. 3B and C). The prognoses of melanoma patients were calculated by the following formula:

2 Riskscore=(−0.1190*PLA2G2Dexpression)+(−0.2133*FUT8expression)+(0.1491*PLA2G4Eexpression)+(−0.2252*PLA2G5expression)+(−0.9248*PLA2G1Bexpression)+(−0.0554*B3GNT2expression)+(−0.1218*ST3GAL5)

### 3.3 Predictive value of the prognostic signature

**Fig. 4:**
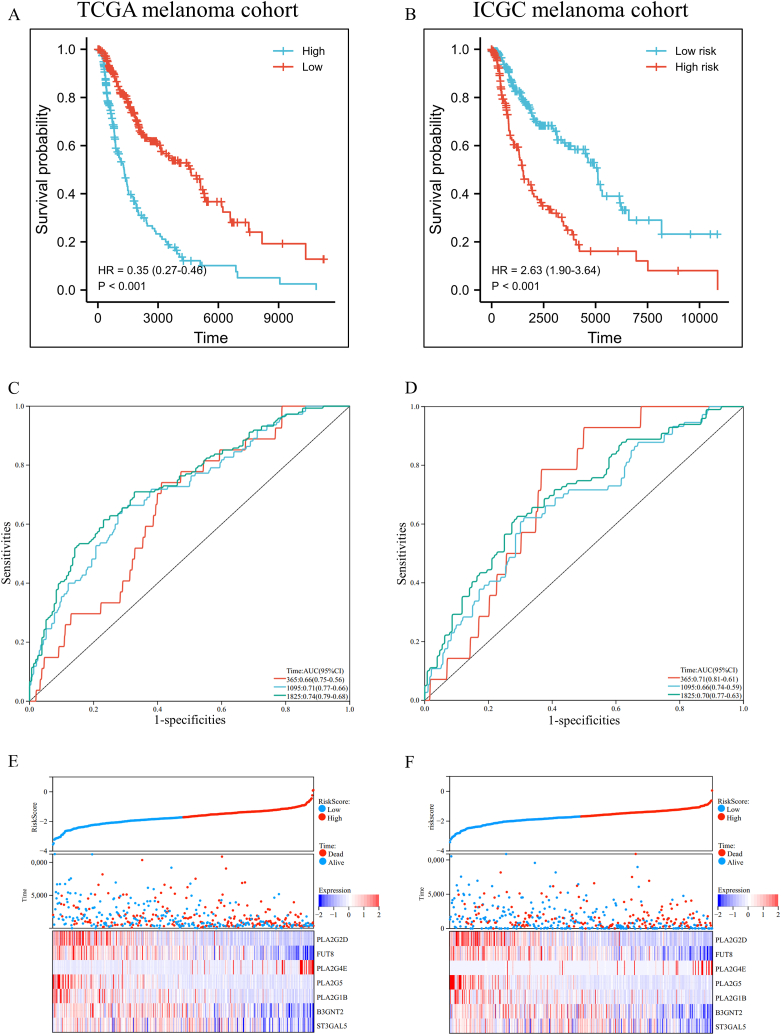
Prognostic analysis of the prognostic signature. (A, B) Kaplan-Meier survival analysis of melanoma patients between high-risk groups and low-risk groups in the TCGA or ICGC cohort. (C, D) Time-dependent ROC curve analysis of the prognostic signature in the TCGA or ICGC cohort. (E, F) The relationship among the risk score, the survival status of patients, and the expression of seven prognostic genes in the TCGA or ICGC cohort.

Melanoma patients in TCGA datasets were stratiﬁed into low-risk and high-risk subgroups based on the optimal cutoff value (−1.633), calculated using the R package maxstat. The melanoma patients in the low-risk subgroups had significantly better survival than those in the high-risk group. In addition, the high-risk group showed a 2.87-fold higher risk (95% confidence interval (CI): 2.19–3.76, *p-value* < 0.001) than the low-risk group (Fig. 4A and E). The area under the ROC curve (AUC) of 1-, 3- and 5-year survival was 0.66, 0.71, and 0.74, respectively (Fig. 4C), indicating good predictive accuracy. To further validate this prognostic signature's versatility and accuracy, the ICGC cohort of melanoma patients was similarly divided into high-risk and low-risk subgroups. Consistent with the TCGA melanoma cohort results, patients in the low-risk group had significantly better survival than those in the high-risk group (Fig. 4B and F). Furthermore, the prognostic signature achieved an AUC of 0.72 at 1 year, 0.67 at 3 years, and 0.70 at 5 years, respectively (Fig. 4D).

### 3.4 Association between the prognostic signature and clinical characteristics

**Fig. 5:**
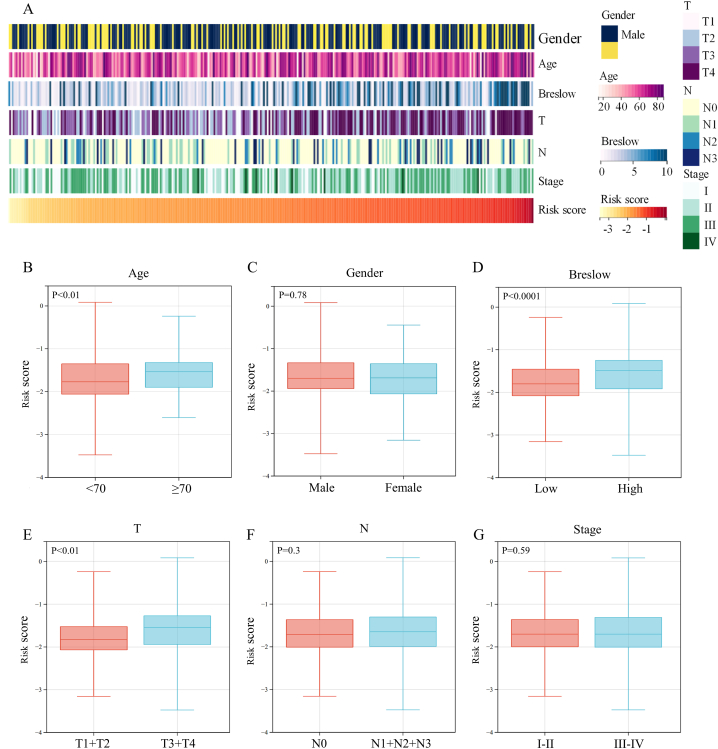
Association between the prognostic signature and clinical characteristics.

**Fig. 6:**
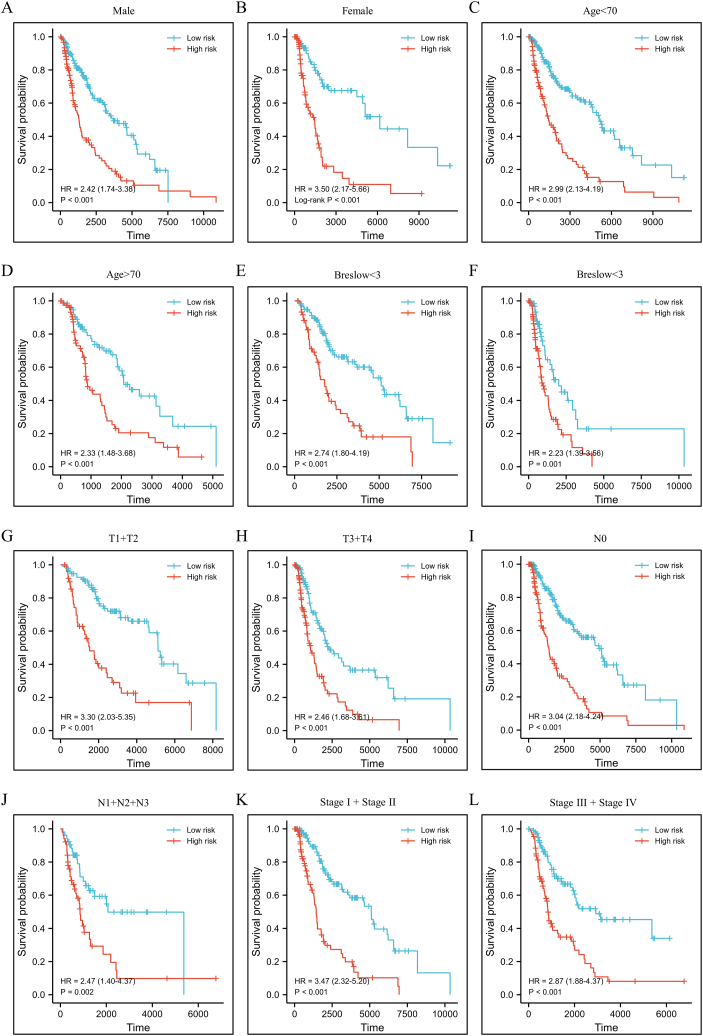
Kaplan-Meier survival analysis of melanoma patients in different subgroups stratified by gender, age, Breslow values, T stage, N stage, or TNM stage.

We compared risk scores between different clinical characteristics subgroups to investigate the association between prognostic signature and clinical characteristics (Fig. 5A). The results showed melanoma patients with higher age, higher Breslow values, and higher T stage had a significantly higher risk score (Fig. 5B, D, and E). However, the risk score did not show any relationship with gender, N stage, or TNM stage (Fig. 5C, F, and G). Further stratification analysis to investigate the significance of the proposed prognostic signature in all subgroups revealed good predictive performance (Fig. 6).

### 3.5 Construction and validation of a nomogram based on the prognostic signature

**Fig. 7:**
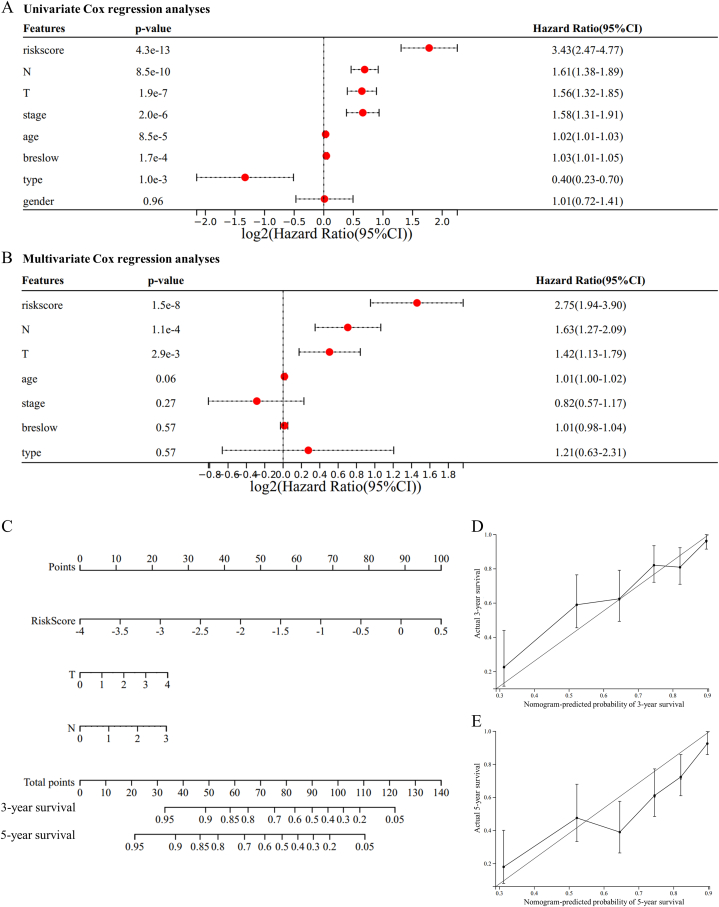
Construction and evaluation of the nomogram. (A, B) Univariate and multivariate regression analysis of the relation between the prognostic signature and clinical variables regarding prognostic value. (C) Nomogram for predicting the probability of 3-, and 5-year OS for melanoma patients. (D) The Calibration plots of the nomogram showed the comparison between the predicted and actual probability of 3-, and 5-year survival.

Univariate and multivariate Cox regression analyses were performed to determine if the prognostic signature's predictive efficacy was independent of other clinical variables in the TCGA melanoma cohort. The prognostic signature continued to be an independent predictive factor after correcting for clinical characteristics such as gender, age, pathologic stage, Breslow depth, and sample type, thus demonstrating its robustness for independently predicting melanoma patients' prognosis (Fig. 7A and B). The multivariate Cox regression analysis indicated that the prognostic signature was independently correlated with the survival information *(p-value* < 0.0001, HR = 2.75, 95% CI = 1.94–3.90). Then, we constructed a nomogram that combined the prognostic signature with independent clinical characteristics (T and N stage) to give clinicians a quantitative method for predicting the prognosis of melanoma patients (Fig. 7C). Consistent with the multivariate Cox regression analysis findings, the prognostic signature contributed the most risk points (Fig. 7C). The calibration plot for the probability of 3 and 5-year OS showed a sufficient overlap between nomogram predictions and actual observations (Fig. 7D and E), indicating a satisfactory predictive accuracy. In addition, the concordance index of the nomogram was 0.738, confirming the favorable prediction ability of the nomogram.

### 3.6 Prognostic signatures are associated with the immune function

**Fig. 8:**
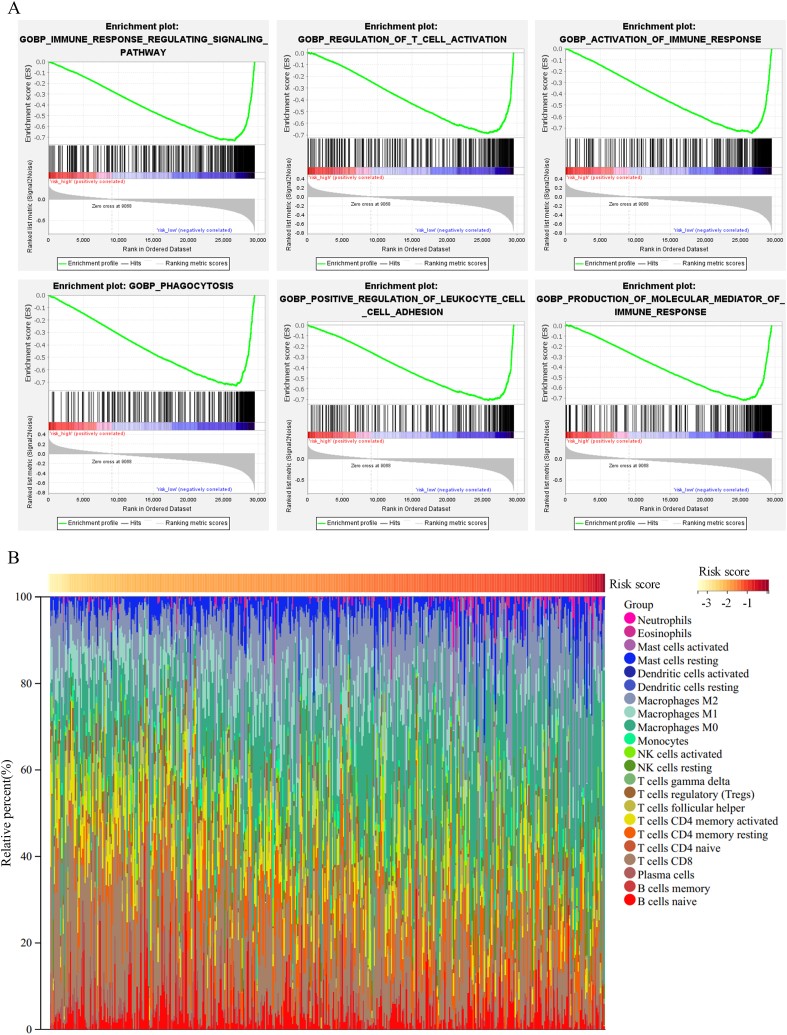
Prognostic signatures are associated with the immune function. (A) Significant enriched immune-related signaling pathways in the low-risk group. (B) The relative proportion of 22 immune cells in melanoma patients.

The underlying molecular mechanisms and biological processes associated with the prognostic signature were elucidated using GSEA. The result showed that immune response regulating signaling pathway (NES = −0.729, size = 472), regulation of T cell activation (NES = −0.686, size = 359), immune response activation (NES = −0.746, size = 367), phagocytosis (NES = −0.730, size = 394), positive regulation of leukocyte cell-cell adhesion (NES = −0.706, size = 266), and molecular mediator of immune response (NES = −0.722, size = 298) were mainly enriched in the low-risk group (Fig. 8A). These signaling pathways are essential for the proper functioning of the immune system due to their contribution to the immune defense and surveillance of tumors. In contrast, immune-related biological processes were hardly enriched in the high-risk group, indicating that the prognostic signature may be a vital predictor for the immune function of melanoma patients.

**Fig. 9:**
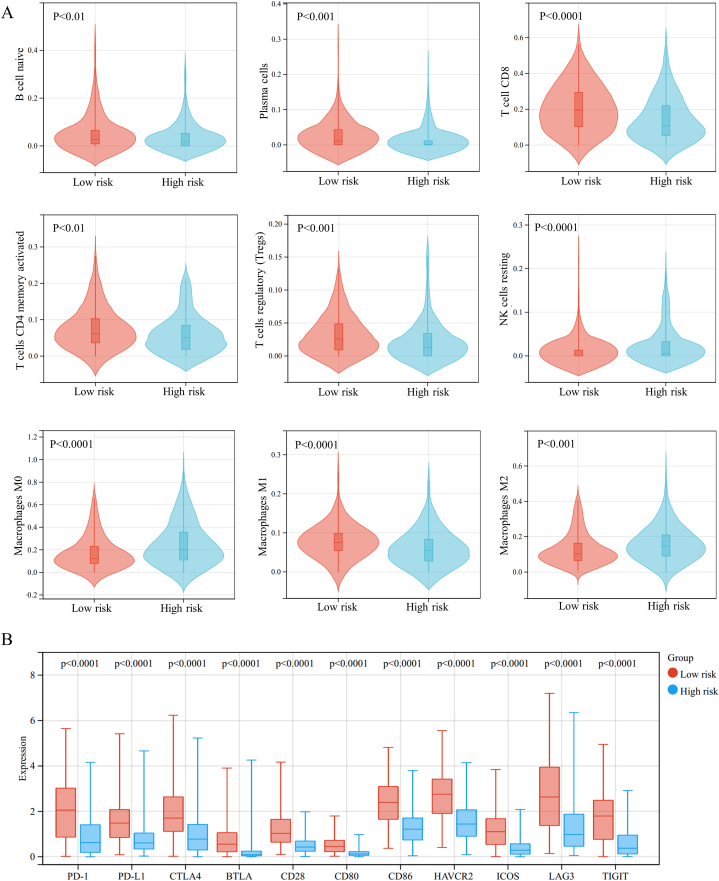
Differences in the immune environment between high-risk and low-risk groups. (A) Violin plots visualizing significantly different immune cells between low-risk and high-risk groups. (B) Box plot showing significantly higher expression of immune checkpoint molecules in the low-risk group.

Considering the possible implications of the prognostic signature in immune function, we estimated the composition of 22 immune cell types in the melanoma samples using CIBERSORT in combination with the LM22 signature matrix (Fig. 8B). The result of CIBERSORT revealed that the low-risk melanoma patients had significantly higher proportions of naive B cells, plasma cells, CD8 T cells, CD4 memory-activated T cells, regulatory T cells, and M1macrophages and considerably fewer resting NK cells, as well as M0 and M2 macrophages than the high-risk melanoma patients (Fig. 9A). In addition, we investigated the correlation between the prognostic signature and key immune checkpoints due to the significance of checkpoint inhibitors in melanoma immunotherapy. The expression of immune checkpoint molecules (PD-1, PD-L1, CTLA4, BTLA, CD28, CD80, CD86, HAVCR2, ICOS, LAG3, and TIGIT) in the low-risk group was significantly higher than that in the high-risk group (Fig. 9B), indicating that the melanoma patients in the low-risk group may have a more favorable response to immune checkpoint inhibition.

## 4 Discussion

Melanoma is the most aggressive form of skin cancer, accounting for approximately 80% of all skin cancer deaths, although it makes up less than 5% of all skin cancer cases [23]. RAS–RAF–MEK–ERK signaling pathway plays an important role in the pathogenesis and progression of melanoma. The BRAF gene mutation correlates to melanoma patients' prognosis, and therefore, further investigation of signaling pathways and genes perturbed by BRAF is necessary [24,25]. Our study established a prognostic model based on the enriched KEGG pathways in the BRAF mutant group, and its predictive accuracy was evaluated by the ROC curve analysis. The results of this study revealed that the melanoma patients in the low-risk group had significantly better OS. The multivariable Cox analyses indicated that the risk score, T stage, and N stage were independent prognostic indicators for melanoma patients. We then constructed a nomogram based on these independent prognostic indicators, and the predictive accuracy was assessed by the calibration curve and C-index. In addition, we found that the prognostic signature was closely related to immune cell infiltration and immune checkpoint molecules were significantly higher in the low-risk group.

Predicting the accurate prognosis of melanoma patients is essential for clinical practice. Earlier prognostic models for melanoma patients were developed based on hypoxia score [13], ferroptosis-related gene [14], immune-related gene [15], glycolysis-related gene [16], and pyroptosis-related lncRNA [17]. It is not appropriate to make definitive comparisons among these studies since different data sources were used to evaluate the prognostic models. To the best of our knowledge, there was no prediction model based on BRAF mutation status in melanoma. Such a model may help further understand the BRAF gene's role in melanoma pathogenesis and improve the OS rate of melanoma patients. Herein, we established a prognostic model based on the BRAF mutant of melanoma patients. As per the ROC analysis, the AUC for predicting 5-year overall survival was 0.74 in the training cohort (TCGA database) and 0.70 in the validation cohort (ICGC database). In addition, a nomogram based on the prognostic model, T stage, and N stage was constructed to help predict the 3- or 5-year survival rate of melanoma patients in clinical settings. However, our mutation analysis showed that the mutation rate of BRAF was less than 2.5% in ocular melanomas (UVM) (Supplementary Fig. 1), indicating that BRAF mutation may have little to do with the development of UVM and our proposed prognostic signature does not apply for non-cutaneous melanoma.

For advanced melanoma patients, the chance of surgical resection is often limited, and few other treatment options exist, resulting in poor prognosis. Several FDA-approved BRAF or MEK inhibitors (vemurafenib, dabrafenib, and trametinib) have shown promise in improving PFS, RR, and OS for patients with metastatic melanoma [9,10,26]. However, the prognosis of melanoma patients with BRAF wild-type has not been significantly improved, as they do not benefit from the BRAF and MEK inhibitor therapy. Drug resistance also limits the clinical application of BRAF and MEK inhibitors [7]. Furthermore, melanoma patients with tandem mutation in BRAF^V600; K601^ were less responsive to combined BRAF/MEK inhibitors [27]. Previous studies have reported that lipid metabolism and bioactive oxylipin metabolism may be implicated in melanoma resistance to BRAF inhibitors [28,29], consistent with our findings that the ether lipid metabolism was enriched in BRAF-mutant melanoma. In this study, we identified enriched pathways and DEGs associated with OS in BRAF-mutant melanoma via RNA sequencing and analysis of clinical survival data from melanoma samples. These results may help guide molecular-based treatment strategies and provide insights into the differential response to targeted therapy among BRAF-mutant and BRAF wild-type melanoma.

To predict the prognosis of melanoma patients, we calculated the risk score of each patient based on the expression and corresponding lambda value of seven genes (PLA2G2D, FUT8, PLA2G4E, PLA2G5, PLA2G1B, B3GNT2, and ST3GAL5). Previous studies have reported that PLA2G2D and ST3GAL5 may be potential tumor biomarkers associated with immune cells [30,31]. In addition, a few other studies revealed that FUT8, PLA2G5, PLA2G1B, and B3GNT2 were involved in the immune response [[32], [33], [34]]. Furthermore, PLA2G4E has also been reported to play an anti-inflammatory role in skin or adipose tissue inflammation [35,36]. Taken together, these results indicate that the prognostic signature based on these seven genes was related to immune cell infiltration and immune checkpoint.

Despite several therapies available for melanoma patients, including chemotherapy, radiation, and surgical resection, advanced melanoma consistently recurs, and the prognosis is still inferior. One of the possible mechanisms is that these treatments severely suppress the immune system and make it easier for tumors to metastasize and escape from immune surveillance [37]. Therefore, there is an increased focus on tumor immunotherapy research. James P. Allison (Professor of the University of Texas MD Anderson Cancer Center) and Tasuku Honjo (Honorary Professor of Kyoto University) were awarded the 2018 Nobel Prize in Physiology or Medicine for their pioneering discovery of cancer therapy by inhibition of negative immune regulation. Specifically, they identified immune checkpoints (CTLA-4 and PD-1) and demonstrated the feasibility of anti-cancer immunotherapy involving antibodies targeting these checkpoints [[38], [39], [40], [41]]. For instance, ipilimumab, a human monoclonal antibody that blocks CTLA-4, was reported to significantly improve the OS, distant metastasis-free survival, and recurrence-free survival for stage III melanoma [42]. Another clinical trial confirmed the significant benefit of nivolumab (monoclonal antibodies targeting PD-1) for advanced BRAF wild-type melanoma [43]. Moreover, a recent study showed that efficacy of the neoantigen nanovaccine combined with *anti*-PD-1 antibody or Treg inhibiting peptide P60 was better than that of the single treatment [44]. The clinical application of these ICIs has significantly improved the prognosis of melanoma patients. In this study, we found higher expression levels of the immune checkpoint molecules (CTLA-4, PD-1, PD-L1, etc.) in the low-risk group, indicating that these melanoma patients may have a more favorable response to ICIs. Finally, our results may provide a reference for future studies on melanoma patients' immunotherapy.

In conclusion, we identified significantly enriched KEGG pathways and corresponding genes in the BRAF mutant group via GSEA. For the first time, a prognostic signature based on seven BRAF-related genes with an independent prognostic value for melanoma patients and related to immune cell infiltration and checkpoint was constructed and validated. Furthermore, we established a nomogram based on the prognostic signature and independent clinical characteristics, providing a tool for the objective assessment of the survival rate of melanoma patients.

**Funding**

This work was supported by National Natural Science Foundation of China (No. 81902796), the Science and Technology Department of Sichuan Province (No. 2022NSFSC1594), the Science and Technology Cooperation Project of Suining First People’s Hospital and Southwest Medical University (No. 2020SNXNYD01), the Scientific Research Foundation of Southwest Medical University（2021ZKZD011）and National Undergraduate Training Programs for Innovation and Entrepreneurship (S202010632176).

## Author contribution statement

Xiang Huang; Wanrong Gou: Performed the experiments; Wrote the paper.

Qinxian Song; Yan Huang; Xian Jiang: Analyzed and interpreted the data.

Xue Bo; Chunlei Wen: Contributed reagents, materials, analysis tools or data.

Jianguo Feng; Hong Gao: Conceived and designed the experiments.

## Data availability statement

The data used to support the findings of this study have been deposited in the 4TU.ResearchData repository (https://doi.org/10.4121/21532917.v1).

## Declaration of competing interest

The authors declare that they have no known competing financial interests or personal relationships that could have appeared to influence the work reported in this paper.
