# Supplementary material for: A BRAF mutation-associated gene risk model for predicting the prognosis of melanoma
Source: Heliyon. 2023 May 2;9(5):e15939. doi: 10.1016/j.heliyon.2023.e15939 (PMC10189240; doi:10.1016/j.heliyon.2023.e15939)
Supplement: Multimedia component 1 [file mmc1.docx]

We obtained somatic mutation data of ocular melanomas (UVM) from the Cancer Genome Atlas (TCGA) database (<https://www.cancer.gov/>) using the University of California at Santa Cruz (UCSC) Xena portal (<http://xena.ucsc.edu/>) [1]. The mutation analysis showed that the mutation rate of BRAF was less than 2.5% in UVM.
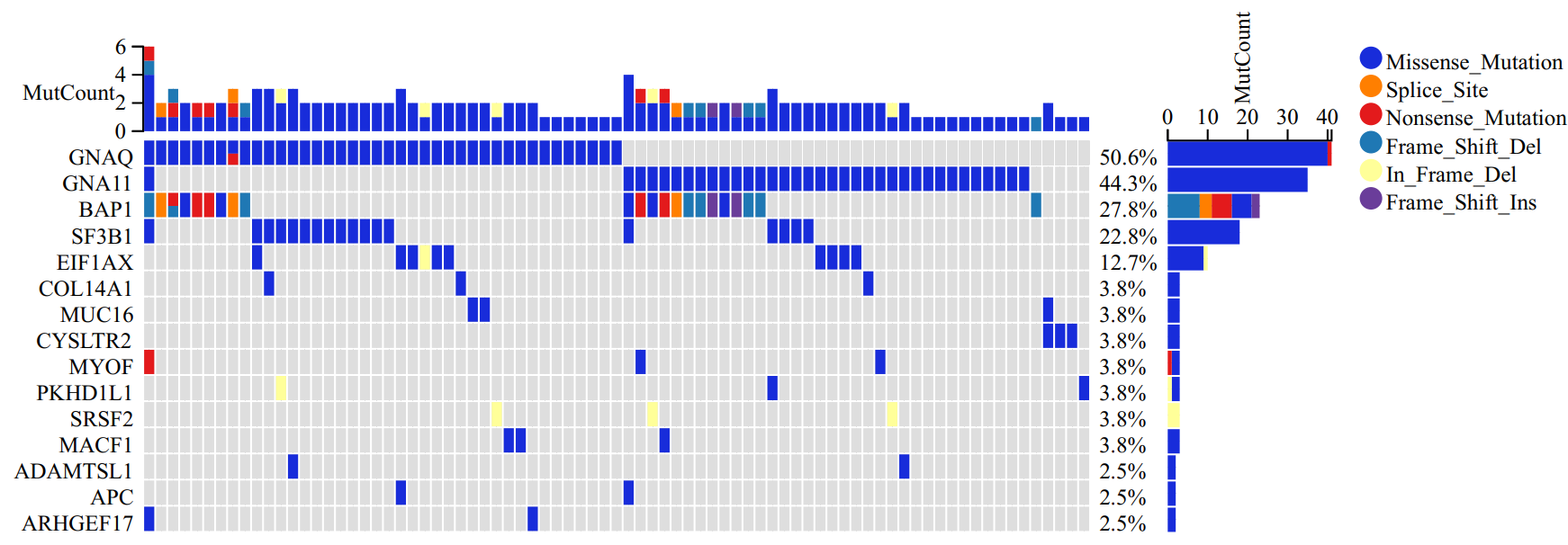
 Supplementary Figure 1. Genomic landscape of ocular melanomas and the mutational signatures in the TCGA cohort.

[1] Goldman, M. J., Craft B., Hastie M., et al., Visualizing and interpreting cancer genomics data via the Xena platform*.* Nat. Biotechnol. 38 (2020) 675-678, <https://doi.org/10.1038/s41587-020-0546-8>
